# Clinical competence, communication ability and adherence to choosing wisely recommendations for lipid reducing drug use in older adults

**DOI:** 10.1186/s12877-023-04429-5

**Published:** 2023-11-20

**Authors:** Robyn Tamblyn, Teresa Moraga, Nadyne Girard, Fiona K. I. Chan, Bettina Habib, John Boulet

**Affiliations:** 1 Department of Epidemiology, Biostatistics and Occupational Health, McGill University, 2001 McGill College Avenue., Montreal, QC H3A 1G1 Canada; 2 Department of Medicine, McGill University Health Center, Montreal, QC Canada; 3 Clinical and Health Informatics Research Group, McGill University, Montreal, QC Canada; 4 Foundation for Advancement of International Medical Education and Research (FAIMER), Philadelphia, PA USA

**Keywords:** Older adults, Lipid-reducing drugs, Choosing wisely

## Abstract

**Background:**

Although lipid-lowering drugs are not recommended for primary prevention in patients 75+, prevalence of use is high and there is unexplained variation in prescribing between physicians. The objective of this study was to determine if physician communication ability and clinical competence are associated with prescribing lipid-lowering drugs for primary and secondary prevention.

**Methods:**

We used a cohort of 4,501 international medical graduates, 161,214 U.S. Medicare patients with hyperlipidemia (primary prevention) and 49,780 patients with a history of cardiovascular disease (secondary prevention) not treated with lipid-lowering therapy who were seen by study physicians in ambulatory care. Clinical competence and communication ability were measured by the ECFMG clinical assessment examination. Physician citizenship, age, gender, specialty and patient characteristics were also measured. The outcome was an incident prescription of lipid-lowering drug, evaluated using multivariable GEE logistic regression models for primary and secondary prevention for patients 75+ and 65-74.

**Results:**

Patients 75+ were less likely than those 65-74 to receive lipid-lowering drugs for primary (OR 0.62, 95% CI 0.59-0.66) and secondary (OR 0.70, 95% CI 0.63-0.78) prevention. For every 20% increase in clinical competence score, the odds of prescribing therapy for primary prevention to patients 75+ increased by 24% (95% CI 1.02-1.5). Communication ability had the opposite effect, reducing the odds of prescribing for primary prevention by 11% per 20% score increase (95% CI 0.8-0.99) for both age groups. Physicians who were citizens of countries with higher proportions of Hispanic (South/Central America) or Asian (Asia/Oceania) people were more likely to prescribe treatment for primary prevention, and internal medicine specialists were more likely to treat for secondary prevention than primary care physicians.

**Conclusion:**

Clinical competence, communication ability and physician citizenship are associated with lipid-lowering drug prescribing for primary prevention in patients aged 75+.

**Supplementary Information:**

The online version contains supplementary material available at 10.1186/s12877-023-04429-5.

Health care spending accounts for approximately 4.6%-18.8% of GDP in OECD countries. Differences in expenditure, ranging from 4.6% in Poland to 18.8% in the United States, are not associated with a commensurate variation in health outcomes [1–4]. One explanation for this lack of relationship, among others such as higher prices in the US [5], is that an estimated one-third of expenditures are for medical services that are unnecessary and possibly harmful [6, 7]. To address this problem, the American Board of Internal Medicine and Consumer Reports launched the Choosing Wisely Campaign, which provided recommendations for the reduction in the use of unnecessary tests and treatments in internal medicine [8]. Since that inaugural event, many medical specialty groups and countries have identified over 300 “do-not-do” low value health care practices [9]. One of the earliest recommendations was to not prescribe lipid lowering drugs for the primary prevention of cardiovascular disease in patients with a limited lifetime expectancy, that is, those 75 years of age or older [10].

The prescription of lipid lowering drugs, specifically the “statins”, has more than doubled in the past decade in many countries [11], with growing evidence of their real-world effectiveness in the prevention of cardiovascular disease. They are now among the top five prescribed medications in North America and Europe [12, 13]. While Choosing Wisely guidelines have advised against prescribing lipid lowering drugs to persons 75 years of age or older, the prevalence of use for primary prevention in this age group is similar to, if not higher than, use in those under the age of 75 [14, 15]. However, there is considerable unexplained variation in adherence to lipid prescribing guidelines between physicians. Similar variations between physicians have been documented in the ordering of unnecessary lab tests, imaging, preventive screening, and antibiotic prescribing [16]. Physician characteristics associated with low value care vary by the indicator used, but common predictors include older and male physicians, larger fee for service vs capitated practices, and urban practice settings with a high specialist to primary care ratio [17–19]. Qualitative studies suggest that low value care may also be more likely to be ordered by physicians who are less knowledgeable, and/or have greater diagnostic uncertainty [20–22]. Communication ability has also been considered critical in order to effectively address patient demand and provide appropriate education. To date, no study has investigated whether these characteristics are associated with variation in the use of low value health care services, nor has any study examined physician characteristics associated with variation in lipid lowering drug prescribing.

We had a unique opportunity to evaluate the contribution of clinical competence and communication ability to variation in lipid prescribing for primary and secondary prevention of cardiovascular disease in older patients above and below the age of 75. We tested the hypothesis that greater clinical competence and better communication ability would be associated with recommended guidelines to only prescribe lipid lowering therapy for primary prevention to persons aged less than 75 years, and to prescribe lipid lowering therapy for secondary prevention in all patients with a prior history of cardiovascular events.

## Methods

### Design

A cohort of international medical graduates (IMGs) who successfully completed the Educational Commission for Foreign Medical Graduates (ECFMG) clinical assessment of competence and communication ability and practiced in the United States was assembled. Lipid lowering drug prescribing for primary and secondary prevention was assessed in older Medicare patients seen by these physicians using Medicare data from 2014-2015.

### Study population

A multi-step process was used to assemble the physician and patient study populations. First, all physicians who completed the required ECFMG clinical assessment examination were identified. Second, physicians were linked by first and last name, sex, and birthdate to the American Medical Association (AMA) Masterfile to identify physicians who had acquired a license to practice in the United States. As assessment and management of cardiovascular risk factors is predominantly conducted by primary care physicians, the population was restricted to physicians in internal medicine, family medicine or general practice, based on the specialty recorded in the AMA Masterfile. Third, the national provider identifier of each physician was used to link to the Center for Medicare and Medicaid Services (CMS) administrative files to identify physicians in the cohort who had billed for Medicare patients. All patients who were 65 years of age or older and seen by these physicians in 2014 and 2015 were identified in the Medicare Carrier RIF file, inpatient files, outpatient file, and Part D files and then all health care services and medications received by these patients by any health professional were retrieved.

Patients were eligible for consideration if they had an evaluation and management visit in an outpatient setting with a study physician between July 2014 and November 2015, had continuous Part D drug coverage, had hyperlipidemia or a history of cardiovascular disease, and had not been dispensed a prescription for a lipid lowering drug from any physician in the 6 months prior to the evaluation visit. Thus, eligible patients were restricted to those whose management was being assumed by the study physician and were not prevalent users of lipid-lowering medications.

To evaluate primary prevention, all patients with hyperlipidemia and no history of cardiovascular disease were identified, and stratified by age: 75+ where primary prevention was not recommended, and 65-74 where treatment with lipid-lowering drugs was recommended. To evaluate secondary prevention, all patients with a history of cardiovascular disease were identified and were also stratified by age (75+ and 65-74) to enable comparisons with primary prevention. The presence of hyperlipidemia was measured using the Medicare chronic disease listing. Cardiovascular disease was defined as a diagnosis of acute myocardial infarction, other acute and subacute forms of Ischemia, old myocardial infarction, angina pectoris, and other forms of chronic ischemic heart disease recorded in visits in the 6 months before the evaluation visit. To enable comparisons of physician characteristics that were associated with prescribing, for primary and secondary prevention, we restricted the physician population to those that conducted evaluation and management services to patients in both cohorts and age groups.

### Outcome: prescription of lipid-lowering medication

We used data in the Part D drug insurance file of lipid-reducing medications dispensed within 30 days of an evaluation or management visit to measure prescription of these medications by the physician with whom the visit took place. Lipid-lowering drugs included HMG CoA reductase inhibitors (statins), cholesterol absorption inhibitors, PCSK9 inhibitors, citrate lyase inhibitors, bile acid sequestrants, fibrates, niacin, and omega-3 fatty acids. The prescribing physician had to be the study physician who conducted the evaluation visit. However, due to primary non-adherence to medications [23], these may not represent all lipid-reducing drug prescriptions written by physicians.

### Predictors

#### Clinical competence and communication ability

The Clinical Skills Assessment (CSA) Examination administered by the ECFMG between 1998 and 2004 was used as a measure of clinical competence. The CSA was put in place to ensure that all IMGs could demonstrate an acceptable level of clinical skills necessary for entry into US graduate medical education programs. The CSA was subsequently replaced by USMLE Step 2 Clinical Skills, which, as of 2004, was required for graduates of all US and foreign medical schools. The CSA consisted of 10 or 11 modeled encounters between the candidate and a standardized patient. An overall *clinical competence score* was given based on history taking and physical examination conducted in these encounters and each candidate’s diagnosis and management plan as written in a post-encounter clinical note. An overall *communication score* was given based on the candidate’s interpersonal skills, assessed in each encounter by the standardized patient, as well as their spoken English proficiency. An acceptable clinical competence and communication score was required to pass the examination.

#### Other physician characteristics

Physician age, sex, specialty, and practice region have been associated with a variety of quality of care indicators [17–19, 24, 25]. These data were retrieved from the ECFMG database and the AMA Masterfile. As cardiovascular risk varies between different races and countries, we hypothesized that the physician’s country of origin may influence both their sensitivity to the importance of cardiovascular risk factor management as well as possibly the mix of patients in their respective practices. Physician citizenship at the time of medical school graduation was obtained from the ECFMG database and grouped into twelve geographic regions.

#### Patient characteristics

Patient characteristics that would influence both cardiovascular risk and the likelihood of lipid drug prescribing may differ between physician practices. For this reason, we measured patient sex and race (White, Black, Asian, Hispanic, NA Native, other) using data from the CMS Master Beneficiary Summary File. For patients with a history of cardiovascular disease, we measured whether there was a diagnosis of hyperlipidemia in the Medicare Chronic Conditions file. Multimorbidity may reduce the likelihood of lipid-lowering drug prescribing if associated with limited life expectancy. We used the Elixhauser index [26, 27] to measure co-morbidities associated with an increased risk of mortality using diagnostic data from the outpatient, inpatient and carrier files in the six months prior to the evaluation and management visit. A count of the number of active medications at the time of the evaluation and management visit was also measured using the Part D files.

### Analysis

Descriptive statistics were used to summarize physician and patient characteristics. To estimate the association between clinical competence, communication ability and the odds of lipid-lowering drug prescribing for primary and secondary prevention, we used GEE logistic regression. Patient was the unit of analysis and physician was the clustering factor, accounted for using an exchangeable correlation coefficient. Clinical competence and communication scores were fit in separate models as continuous variables, adjusting for other physician and patient characteristics. Separate models were created for the primary prevention and secondary prevention cohorts. To test the hypothesis that clinical competence and communication ability would reduce the odds of lipid-drug prescribing for primary prevention in patients 75+, but not for secondary prevention, we added a two-way interaction term between score and age group in the primary prevention and secondary prevention cohort models. All analyses were done using SAS version 9.4.

## Results

**Fig. 1:**
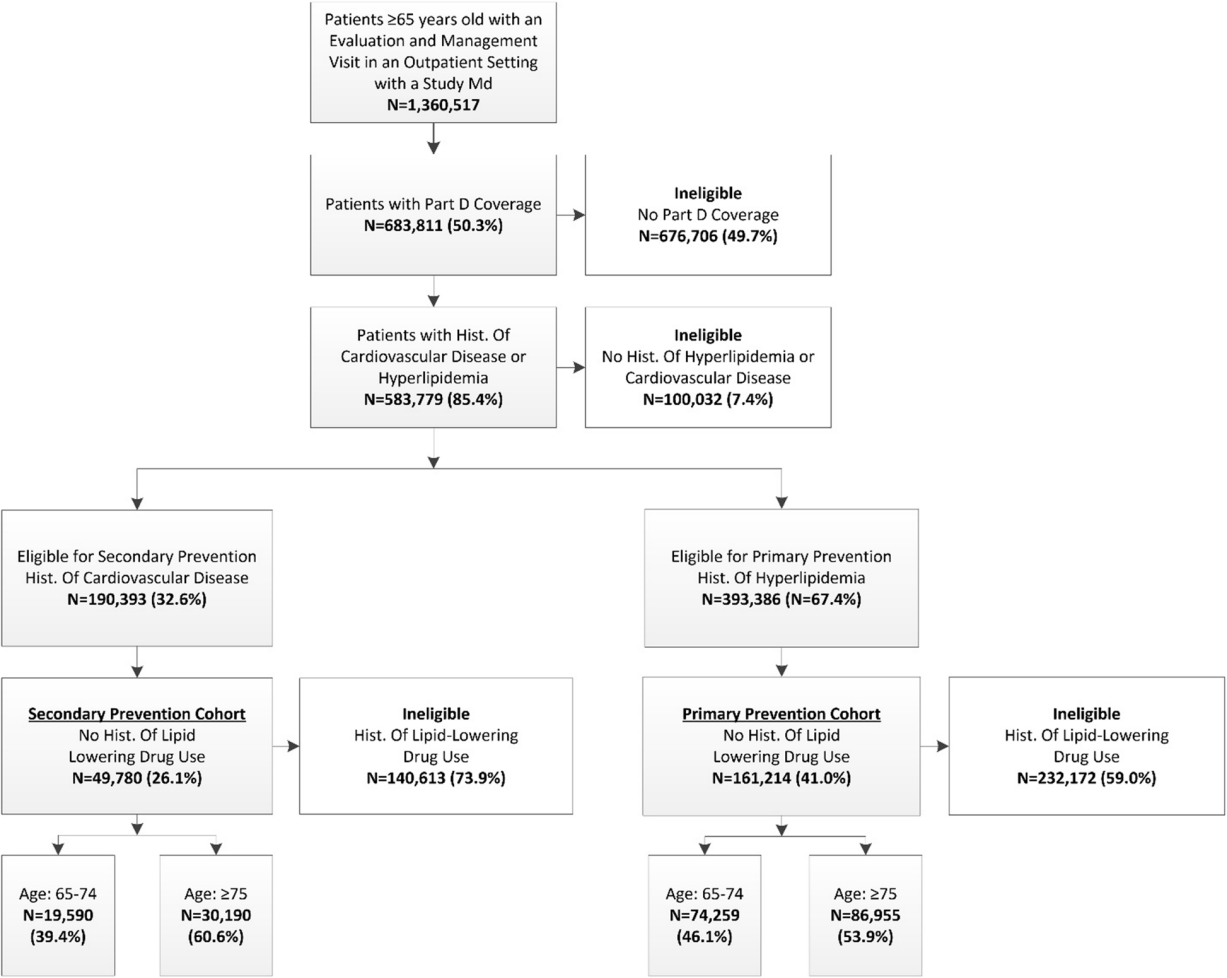
Medicare patients seen by study physicians who were eligible for the primary and secondary prevention cohort

Among the 32,908 physicians who successfully completed the ECFMG examination, 26,023 applied for a license to practice and were found in the AMA files, 9314 of whom were in family medicine, general practice or internal medicine, and 4,501 (70.5%) billed for patients in both age groups in the primary and secondary prevention cohorts (supplement-figure 1). Overall, 60.5% of these 4,501 physicians were male, with a mean age of 43.4 years (Table 1). Approximately one-quarter were citizens of India at the time of medical school graduation (26.2%), 18% were citizens of the United States, and 13.2% were from Asia. 60.9% specialized in internal medicine, and 39.4% practiced in the southern U.S. Mean scores for communication ability were higher but more variable (77.38±7.91) than clinical competence scores (64.32±5.28).

**Table 1:** The Characteristics of the 4,501 physicians who conducted an evaluation and management visit in each of the four medicare cohorts

**Characteristic**	**N (%)**
**Physician Gender**	
Female	1,779 (39.5%)
Male	2,722 (60.5%)
**Citizenship**	
Africa	309 (6.9%)
Canada	38 (0.8%)
Eastern Europe	325 (7.2%)
Europe	105 (2.3%)
India	1,180 (26.2%)
Mexico/Central America/Caribbean	203 (4.5%)
Middle East	388 (8.6%)
Oceania/Asia	596 (13.2%)
Pakistan	343 (7.6%)
South America	150 (3.3%)
United Kingdom	54 (1.2%)
United States	810 (18.0%)
**Physician Specialty**	
Primary care	1,762 (39.2%)
Internal Medicine	2739 (60.8%)
**Region of Practice**	
Northeast	936 (20.8%)
Midwest	930 (20.7%)
South	1,775 (39.4%)
West	860 (19.1%)
	**Mean (SD)**
**Physician Age**	43.4 (5.5)
**Clinical Skills Assessment**	
Clinical Competence	64.3 (5.3)
Communication Score	77.4 (7.9)

Study physicians billed for an evaluation and management visit for 1,360,517 Medicare patients aged 65 or older in an ambulatory visit between July 2014 and November 2015; 583,779 of patients had Part D drug coverage, a history of cardiovascular disease or hyperlipidemia and were potentially eligible for inclusion (Fig.  1). Among patients with a history of cardiovascular disease, 140,613 (73.9%) were prevalent users of lipid-lowering drugs. For those with hyperlipidemia alone, 232,172 (59.0%) were prevalent users. The remaining 161,214 patients were included in the primary prevention cohort and 49,780 were included in the secondary prevention cohort (Table 2).

**Table 2:** Characteristics of patients in the primary and secondary prevention cohorts in the 6 months prior to the evaluation and management visit, and the incidence of lipid drug prescribing

Characteristic	Primary Prevention Cohort Only Hyperlipidemia (*N*=161,214)		Secondary Prevention Cohort History of Cardiovascular Disease (*N*=49,780)	
	75+ Years Old (*N*=86,955 )	65-74 Years Old (*N*=74,259)	75+ Years Old (*N*=30,190)	65-74 Years Old (*N*= 19,590 )
	Mean (SD)	Mean (SD)	Mean (SD)	Mean (SD)
Patient Age	83.0 (5.8)	69.9 (2.6)	83.8 (5.8)	69.8 (2.7)
Patient Gender	N(%)	N(%)	N(%)	N(%)
Male	23,703 (27.3%)	24,442 (32.9%)	12,123 (40.2%)	9357 (47.8%)
Female	63,252 (72.7%)	49,817 (67.1%)	18,067 (59.8%)	10233 (52.2%)
Patient Race				
White	71,113 (81.8%)	59,231 (79.8%)	24,784 (82.1%)	14938 (76.3%)
Black	7,269 (8.4%)	7,776 (10.5%)	2,743 (9.1%)	2938 (15.0%)
Asian	3,056 (3.5%)	2,044 (2.8%)	746 (2.5%)	396 (2.0%)
Hispanic	3,601 (4.1%)	2,567 (3.5%)	1,328 (4.4%)	656 (3.4%)
Native American	264 (0.3%)	246 (0.3%)	114 (0.4%)	100 (0.5%)
Other race	1,652 (1.9%)	2,395 (3.2%)	475 (1.6%)	562 (2.9%)
Elixhauser Index	N (%)			
0 comorbidity	7,825 (9.0%)	14,097 (19.0%)	1,373 (4.6%)	2359 (12.0%)
1-2 comorbidities	22,668 (26.1%)	21,888 (29.5%)	2,268 (7.5%)	1956 (10.0%)
3-4 comorbidities	24,923 (28.7%)	19,302 (26.0%)	5,575 (18.5%)	3791 (19.4%)
+5 comorbidities	31,539 (36.3%)	18,972 (25.6%)	20,974 (69.5%)	11484 (58.6%)
Number Medications				
0 meds	15,384 (17.7%)	15,865 (21.4%)	3,410 (11.3%)	3239 (16.5%)
1-3 meds	36,407 (41.9%)	32,715 (44.1%)	9,213 (30.5%)	6206 (31.7%)
4-5 meds	17,579 (20.2%)	12,783 (17.2%)	6,730 (22.3%)	3865 (19.7%)
+6 meds	17,585 (20.2%)	12,896 (17.4%)	10,837 (35.9%)	6280 (32.1%)
Hyperlipidemia				
yes	86,955 (100.0%)	74,259 (100.0%)	27628 (91.5%)	15870 (81.0%)
no	N/A	N/A	2562 (8.5%)	3720 (19.0%)
Lipid-Lowering Drug Rx				
yes	2,178 (2.5%)	3,195 (4.3%)	840 (2.8%)	815 (4.2%)
no	84,777 (97.5%)	71,064 (95.7%)	29350 (97.2%)	18775 (95.8%)

Patient characteristics varied by cohort (Table 2). The primary prevention cohort was younger and had a higher proportion of females than the secondary prevention cohort, particularly among those aged 75+. The majority of patients were white in both cohorts and age groups (76.3%-82.1%). Patients in the secondary prevention cohort had higher levels of co-morbidity: 58.6%-69.5% had five or more co-morbidities, 32.1%-35.9% were taking six or more medications, and 81.1%-91.5% had hyperlipidemia. Overall the incident prescription of a lipid-lowering drugs was systematically lower for patients 75+ in both the primary prevention (2.5% vs 4.3% for aged 65-74), and secondary prevention cohorts (2.78% vs. 4.16% for aged 65-74).

More clinically competent physicians were more likely to prescribe lipid-lowering drug for primary prevention but only to patients aged 75+ (significant score*age stratum interaction: *p*=0.03) (Table 3). For every 20% increase in clinical competence score, the odds of prescribing a lipid-lowering drug for patients 75+ increased by 24% (OR 1.24, 95% CI 1.02-1.50). Clinical competence was not significantly associated with the odds of prescribing for secondary prevention although the direction and magnitude of the association was similar (OR 1.19, 95% CI 0.96-1.49). Communication ability had the opposite effect on drug prescribing, reducing the odds of prescribing lipid-lowering drugs for primary prevention among patients 65+ by 11% per 20% increase in score (OR 0.89, 95% CI 0.80-0.99), and by 12% (OR 0.88, 95% CI 0.75-1.02) for secondary prevention in patients 65+, although the latter was not statistically significant. There was no significant interaction between communication score and age stratum.

**Table 3:** The association between clinical competence, communication ability and other physician and patient characteristics and the prescription of lipid reducing agents for primary and secondary prevention in patients 75+ and 65-74 years

	**Primary prevention**		**Secondary prevention**	
	**OR (95% CI)**	***P*-Value**	**OR (95% CI)**	***P*-Value**
**Physician Characteristics**				
**Clinical Competence + Communication Scores (per 2 deciles)**				
Overall Competence^a^	N/A	N/A	1.19 (0.96 – 1.49)	0.112
Patients 75+ with Hyperlipidemia	1.24 (1.02 – 1.50)	0.027	N/A	N/A
Patients 65-74 with Hyperlipidemia	0.98 (0.82 – 1.17)	0.784	N/A	N/A
Communication	0.89 (0.80 – 0.99)	0.035	0.88 (0.75 – 1.02)	0.095
**Gender**				
Male	Ref	Ref	Ref	Ref
Female	1.00 (0.92 – 1.08)	0.925	1.04 (0.91 – 1.17)	0.588
**Age (per 5 years)**	1.03 (0.99 – 1.06)	0.124	1.08 (1.02 – 1.13)	0.005
**Citizenship**				
US	Ref	Ref	Ref	Ref
Africa	1.06 (0.86 – 1.30)	0.596	0.80 (0.61 – 1.05)	0.108
Canada	0.91 (0.52 – 1.59)	0.742	0.71 (0.37 – 1.38)	0.318
Eastern Europe	1.25 (1.04 – 1.50)	0.016	0.96 (0.74 – 1.25)	0.724
Europe	1.09 (0.82 – 1.44)	0.575	0.59 (0.34 – 1.02)	0.057
India	1.10 (0.97 – 1.24)	0.150	0.96 (0.80 – 1.16)	0.677
Mexico/Central America/Caribbean	1.33 (1.11 – 1.59)	0.002	1.01 (0.76 – 1.33)	0.964
Middle East	1.07 (0.90 – 1.26)	0.434	0.93 (0.73 – 1.19)	0.567
Oceania/Asia	1.19 (1.03 – 1.38)	0.015	0.92 (0.74 – 1.14)	0.438
Pakistan	1.14 (0.95 – 1.36)	0.171	0.83 (0.64 – 1.07)	0.148
South America	1.36 (1.09 – 1.69)	0.007	1.15 (0.85 – 1.55)	0.375
United Kingdom	0.85 (0.64 – 1.14)	0.286	0.81 (0.46 – 1.44)	0.474
**Specialty**				
Internal Medicine	1.02 (0.94 –1.11)	0.620	1.19 (1.05 – 1.35)	0.008
Primary Care	Ref	Ref	Ref	Ref
**Region of Practice**				
Northeast	Ref	Ref	Ref	Ref
Midwest	1.22 (1.07 – 1.39)	0.003	1.16 (0.96 – 1.40)	0.127
South	1.27 (1.13 – 1.42)	<0.001	1.28 (1.08 – 1.51)	0.004
West	1.15 (1.01 – 1.31)	0.031	1.33 (1.10 – 1.60)	0.003
**Patient Characteristics**				
**Age Stratum**				
+	0.62 (0.59 – 0.66)	<0.001	0.70 (0.63 – 0.78)	<0.001
65-74	Ref	Ref	Ref	Ref
**Gender**				
Male	Ref	Ref	Ref	Ref
Female	0.92 (0.87 – 0.97)	0.003	0.91 (0.83 – 1.00)	0.06
**Race**				
White	Ref	Ref	Ref	Ref
Black	1.35 (1.24 – 1.48)	<0.001	1.17 (1.00 – 1.37)	0.054
Asian	1.92 (1.68 – 2.20)	<0.001	1.68 (1.25 – 2.26)	<0.001
Hispanic	1.82 (1.60 – 2.08)	<0.001	1.60 (1.29 – 1.99)	<0.001
North American Native	1.16 (0.72 – 1.88)	0.534	0.61 (0.24 – 1.56)	0.302
Other	1.51 (1.31 – 1.75)	<0.001	1.60 (1.20 – 2.12)	<0.001
**Weighted Elixhauser**	0.96 (0.95 – 0.97)	<0.001	0.92 (0.91 – 0.94)	<0.001
**Number of active drugs**	0.92 (0.91 – 0.93)	<0.001	0.94 (0.93 – 0.96)	<0.001
**Hyperlipidemia**				
No	N/A		Ref	Ref
Yes	N/A		1.70 (1.45 – 2.00)	<0.001

*Abbreviations*: *OR* odds ratio, *CI* Confidence Interval, *Ref* Reference group, *US* United States

^a^Interaction term between clinical competence score and patient age stratum in primary prevention was significant

Physician citizenship at time of medical school graduation was associated with lipid-lowering drug prescribing for primary prevention but not secondary prevention (Table 3). Compared to U.S. citizens, physicians from South America (OR 1.36, 95% CI 1.09-1.69), Central America (OR 1.33, 95% CI, 1.11-1.59), Asia (OR 1.19, 95% CI 1.03-1.38), and Eastern Europe (OR 1.25, 95% CI 1.04-1.50) were more likely to prescribe lipid-lowering drugs to patients with hyperlipidemia. Compared to physicians practicing in the northeast, those practicing in all other regions were more likely to prescribe lipid-lowering drugs for primary and secondary prevention. Physician age and specialty influenced prescribing for secondary but not primary prevention. Older physicians (OR 1.08 per 5 years, 95% CI 1.02-1.13) and internal medicine specialists compared to primary care (OR 1.19, 95% CI 1.05-1.35) were more likely to prescribe lipid-lowering drugs.

Patient characteristics influenced the likelihood of lipid-lowering drug prescribing in a comparable way for both primary and secondary prevention (Table 3). Patients 75+ were 38% (OR 0.62, 95% CI 0.59-0.66) less likely to receive a lipid-lowering drug for primary prevention and 30% (OR 0.70, 95% CI 0.63-0.78) less likely to receive drug treatment for secondary prevention compared to those aged 65-74. The odds of receiving a lipid-lowering medication were higher with a co-existing diagnosis of hyperlipidemia in those with a cardiovascular history, and in Black, Asian, and Hispanic patients compared to White patients. In contrast, the likelihood of receiving a prescription decreased by 4%-8% for every one point increase in the Elixhauser comorbidity score (OR 0.96, OR 0.92), and by 6%-8% for every one medication increase in the number of active drugs (OR 0.92, OR 0.94) for primary and secondary prevention respectively. Female patents were 8% less likely to receive a prescription compared to males for primary prevention (OR 0.92, 95% CI 0.87-0.97) and 9% less likely for secondary prevention (OR 0.91, 95% CI 0.83-1.00).

## Discussion

This is the first opportunity to examine the role of clinical competence and communication ability in the recommended and non-recommended prescription of lipid-lowering drugs for primary and secondary prevention of cardiovascular disease. We found more competent physicians were more, not less likely, to prescribe lipid-lowering drugs for primary prevention to patients 75+. Greater communication ability was associated with a lower likelihood of prescribing lipid-lowering treatment for primary prevention. Physician citizenship, specialty, age and region of practice were associated with lipid-lowering drug prescribing. Female patients and those with a greater number of comorbidities and drugs were less likely to receive lipid-lowering drugs. Black, Asian and Hispanic patients were more likely than white patients to receive drug treatment.

Physician performance on standardized licensure/certification examinations of clinical competence and communication ability have been shown to predict the likelihood of complaints to licensing authorities, and the quality of prescribing and preventive care, even after years in practice [28–30]. Communication ability played a surprisingly important role in reducing the odds of lipid-lowering drug prescribing for primary prevention. It is possible that physicians with better interpersonal skills opted to intervene by counselling changes in diet and lifestyle, rather than prescribing. The quality of a physician’s interpersonal skills may also be associated with a more productive doctor-patient relationship where treatment options are more likely to be discussed, or alternately with greater skill in inspiring health behavior change through motivational interviewing [31–33]. Better communication skills are associated with a reduction in the likelihood of prescribing unnecessary medication [34–36], but this relationship has not been studied in situations where medication management might be recommended, and should be addressed in future research. Contrary to expectation, greater levels of clinical competence increased, not decreased, the likelihood of lipid-lowering drug prescribing for primary prevention for patients 75+. Higher scores were also associated with a greater likelihood of recommended drug treatment for secondary prevention, although not significant, suggesting that guideline adherence may not be nuanced to situations that are of lower value. Assessments of clinical competence are designed to measure positive actions with respect to the quality of data collection, diagnosis, and management, and would not generally penalize candidates for ordering unnecessary test and treatments [37, 38].

The importance of physician citizenship in predicting treatment for primary prevention of cardiovascular disease has not been previously investigated. We found that physicians who were citizens of countries with a higher proportion of persons who were of Hispanic or Asian origin were more likely to prescribe drug treatment for primary prevention. Of interest, physician citizenship was only a factor in treatment decisions for primary not secondary prevention. Physicians from these countries may be more sensitive to underlying differences in risk of cardiovascular disease in Hispanic, Asian and Black populations. Racial mix and higher levels of cardiovascular risk factors may also explain the regional differences in lipid-lowering drug prescribing with higher levels of cardiovascular risk factors, particularly in the South [39–41]. Consistent with this hypothesis, patients with higher cardiovascular risk, males [42, 43], patients with hyperlipidemia [44, 45], and those who were Black, Asian or Hispanic [46, 47] were more likely to be prescribed a lipid-lowering drug. Also consistent with Choosing Wisely guidelines, those with a lower life expectancy, older patients with multiple comorbidities and drugs were less likely to be prescribed lipid-lowering therapy.

Our finding that internal medicine specialists were more likely than primary care physicians to prescribe lipid-lowering drugs for secondary prevention may be related to a higher concentration of patients with cardiovascular disease in their practice, or greater familiarity with guidelines for secondary prevention. Similar trends are noted in other studies that have compared clinical guideline adherence for specialists in comparison to primary care physicians [48–53].

There are limitations to be considered in the interpretation of the results. First, we measured what medications were dispensed, not what was prescribed. Primary non-adherence to the prescription of a new drug is estimated to be approximately 15% and is as high as 20% for lipid-lowering drugs [23]. As non-adherence has been associated with lower scores on clinical skills examination [54], our study may be measuring a combination of both the likelihood of prescribing lipid-lowering drugs as well as primary adherence to treatment. Our results, based on statin prescribing in 2014-1015, may not represent the incidence of statin prescribing in 2023 when most drugs were off-patent, however this is unlikely to be differential among physicians with varying levels of ability. Also, Choosing Wisely recommendations started in 2011, and the recommendation related to lipid-reducing drug prescribing for primary prevention may not have yet had an impact on training programs of physicians in this cohort. Administrative databases have known limitations in the lack of availability of patient lifestyle and clinical data, characteristics that could influence the likelihood of prescribing and differ among physicians [55–58]. We have no reason to believe that this possibility of residual confounding would be differential among physicians with varying levels of competence and communication ability.

In conclusion, clinical competence and communication ability are associated with the likelihood of prescribing lipid-lowering drug treatment for primary prevention, as is physician citizenship and practice region. Future research should test the hypothesis that physicians with better communication skills are more likely to provide lifestyle and behavioral interventions for patients with hyperlipidemia, and investigate why more clinically competent physicians and those from countries with a higher proportion of the population at risk of cardiovascular disease are more likely to prescribe lipid-lowering drugs for primary prevention.

### Supplementary Information

**Additional file 1:** **eFigure 1.** International Medical Graduates Who Completed the Educational Commission for Foreign Medical Graduates (ECFMG) Clinical Assessment Examination and Were Eligible for the Primary and Secondary Prevention Cohorts.

## Data Availability

The data analyzed in this study are not publicly available. Those wishing to obtain the data used in this study should contact Dr. John Boulet (bouletjr@gmail.com), the data custodian for the Educational Commission for Foreign Medical Graduates, as well as the Centre for Medicare & Medicaid Services DUA team (DataUseAgreement@cms.hhs.gov re: project number DUA RSCH-2017-51585) to obtain approval for access.

## Abbreviations

IMG: International Medical Graduate
ECFMG: Educational Commission for Foreign Medical Graduates
AMA: American Medical Association
CMS: Center for Medicare and Medicaid Services
CSA: Clinical Skills Assessment
OR: Odds Ratio
CI: Confidence Interval
SD: Standard Deviation
