# Supplementary material for: Clinical competence, communication ability and adherence to choosing wisely recommendations for lipid reducing drug use in older adults
Source: BMC Geriatr. 2023 Nov 20;23:761. doi: 10.1186/s12877-023-04429-5 (PMC10662284; doi:10.1186/s12877-023-04429-5)

**eFigure 1.** International Medical Graduates Who Completed the Educational Commission for Foreign Medical Graduates (ECFMG) Clinical Assessment Examination and Were Eligible for the Primary and Secondary Prevention Cohorts


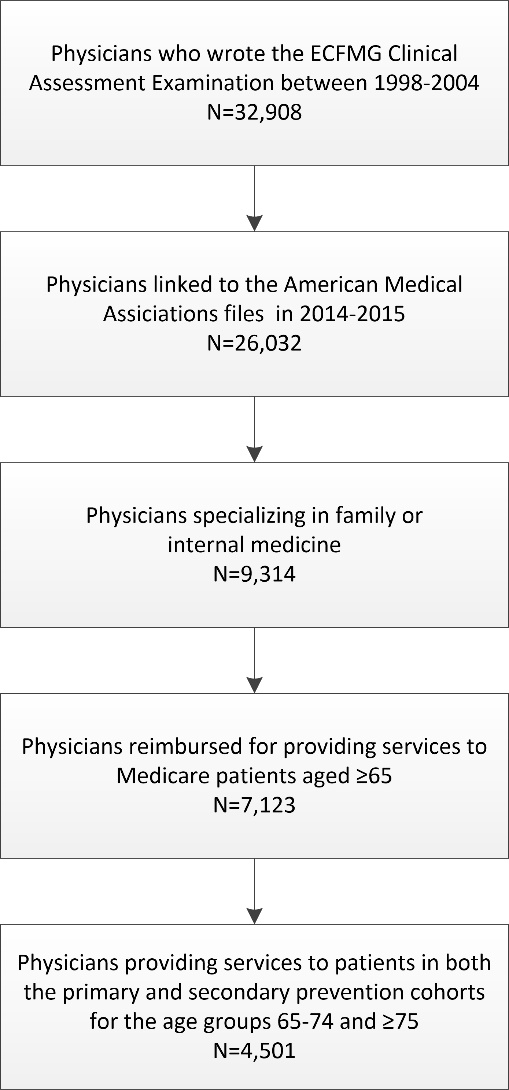

Supplement: Supplementary file 1 — Additional file 1: eFigure 1. International Medical Graduates Who Completed the Educational Commission for Foreign Medical Graduates (ECFMG) Clinical Assessment Examination and Were Eligible for the Primary and Secondary Prevention Cohorts. [file 12877_2023_4429_MOESM1_ESM.docx]
